# Differences in Hygiene Habits among Children Aged 8 to 11 Years by Type of Schooling

**DOI:** 10.3390/children9020129

**Published:** 2022-01-19

**Authors:** Ana María Pérez Pico, Esther Mingorance Álvarez, Julia Villar Rodríguez, Raquel Mayordomo Acevedo

**Affiliations:** 1 Department of Nursing, Universidad de Extremadura, 10600 Plasencia, Cáceres, Spain; aperpic@unex.es (A.M.P.P.); emingorance@unex.es (E.M.Á.); 2 Department of Anatomy Cell Biology and Zoology, Universidad de Extremadura, 10600 Plasencia, Cáceres, Spain; juliavr@unex.es

**Keywords:** habits, hygiene, learning, interdisciplinary practices, foot

## Abstract

Personal hygiene is one of the basic activities in the care of our body. Parents are responsible for their children’s hygiene to prevent infections and keep them healthy. However, children must acquire hygiene habits correctly and independently. This study examines the sociodemographic profile, hygiene habits and knowledge, and level of autonomy of children who are starting to perform their personal care autonomously to identify the areas in which their habits could be improved. A descriptive cross-sectional study was conducted concerning 125 children aged 8–11 years attending schools in northern Extremadura, Spain. The children were surveyed with the HICORIN^®^ questionnaire and the resulting data were statistically processed with SPSS 22.0 (IBM, Armonk, NY, USA). The majority of participating children required help to perform personal hygiene activities. Children in preferential schooling (PS) require less help than children in mainstream schooling (MS) but have less knowledge about personal hygiene. Different habits were observed in the frequency and time of day for performing personal hygiene between groups (*p*-values < 0.005). In general, more than 80% of children aged 8 to 11 years are not autonomous in some aspect of their personal hygiene, and they are not all familiar with personal hygiene. Because of this, it is necessary to conduct theory and practical workshops with children who must acquire correct personal hygiene habits autonomously to prevent infection and promote health.

## 1. Introduction

Hygiene (H) can be defined as the process by which a person cares for their health by cleaning and caring for their body [1,2,3]. From the time a child is born, its parents are responsible for its hygiene, not only to ensure the newborn child feels good but also because it is understood as necessary for good health and to prevent infection [4]. However, children must acquire hygiene habits independently [3,5]. The people involved in the acquisition of these habits include not only parents and family members, who are responsible for teaching basic hygiene, but also health professionals and teachers [1,3,5]. In some cases, family members responsible for instructing a child about the importance of hygiene do not have adequate health knowledge, as many are from a rural context or different cultures and ethnic groups and lack proper instruction themselves or, in particular, have no clear strategy for explaining the importance of hygiene and ensuring it is correctly performed [6]. In the context of increasing diversity in Spanish schools, all pupils must, by law, receive an equal education [7,8]. Schools providing preferential education (PS) enrol a large number of pupils who have specific educational needs due to social, economic, and/or cultural factors [8,9]. Because of this, these schools receive more material and staffing resources. However, authors such as Franco García (2017) consider that “the programs designed for the specific attention of immigrant students in the United States in their attempt to “attend” to their needs, exclude them” [10]. In the classroom, teachers reinforce hygiene habits using games, cards, or activities associated with hygiene [1,4,11,12] and address more serious health problems that can result from a lack of hygiene [13]. In some cases, health professionals also teach pupils how to prevent illness by ensuring adequate hygiene habits [2,14].

In our role as health and education professionals, we analysed participants’ general profile, the aspects in which they need help, and their knowledge and habits with regard to personal hygiene, in two groups of children in the same school year in different types of schooling (mainstream and preferential). We also analysed the possible influence of gender on these aspects.

## 2. Materials and Methods

### 2.1. Legal Documents

Permission was sought from the bioethical Committee of the University of Extremadura, Reference (115/2018), and from the parents of the participating children through the administration of the schools where the study was conducted. Permission was obtained from the author of the validated survey HICORIN^®^ [15] due to copyright.

### 2.2. Methodology

**Figure 1:**
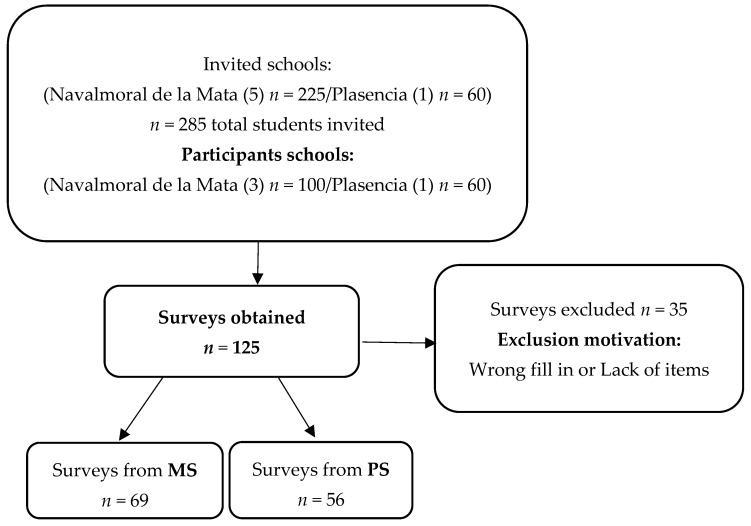
Flowchart of the sample selection. *n*: number; MS: Mainstream Schooling; PS: Preferential Schooling.

A cross-sectional descriptive study was conducted from 14 January 2019 to 14 March 2020 to assess general hygiene habits and hygiene habits specific to the feet. The following flowchart (Figure 1) explains the sampling method.

#### 2.2.1. Sample

Participants comprised 125 school children aged 8 to 11 years (9.37 ± 1.044) enrolled in the fifth year of primary education at schools in Extremadura.

#### 2.2.2. Inclusion and Exclusion Criteria

The inclusion criteria were as follows: be enrolled at a school in Extremadura in the fifth year of primary, be aged 8–11 years, have parents’ authorisation to participate in the study, and fill in all sections of the HICORIN^®^ survey. All participants were also required to be present in the classroom on the day of the survey.

The exclusion criteria were: not having parents’ authorisation to participate in the study and not answering all sections of the HICORIN^®^ survey correctly.

#### 2.2.3. Procedure Followed

Before the study began, parents received an explanation about the purpose of the questionnaire, and the school administration asked them to authorise their children to take part. Parents were also asked about their socioeconomic situation. Once permission had been obtained, the children filled in the validated questionnaire on hygiene habits (HICORIN^®^). The questionnaire comprised 63 questions to analyse four sections: family and sociodemographic characteristics; autonomy; knowledge of hygiene; and hygiene habits. Seven aspects were studied: Hygiene (H); General H; Body and hair H; Hand H; Oral H; Ear H; Intimate H and Foot H.

#### 2.2.4. Statistical Analysis of Data

The data obtained from the questionnaire were analysed using the statistical program SPSS 22.0 (IBM, Armonk, NY, USA). The comparisons between the frequencies obtained in the questions analysed in each aspect of hygiene were analysed using the Chi-square test or Fisher’s Exact Test when more than 25% of cells had an expected frequency of less than 5.

## 3. Results

### 3.1. Analysis of Sociodemographic Characteristics of Participants and Families

Among the participating children, 55.2% were in mainstream schooling and 44.8% in preferential schooling, and 51.2% were girls and 48.8% were boys. Their mean age at the time of the study was 9.37 ± 1.044; the girls were older than the boys (9.41 compared to 9.33 mean age). Most participants were born in Spain (93.6%), as were their fathers (76.8%) and mothers (72.8%). The main foreign countries of origin of their parents were Morocco (father 16.0%; mother 16.8%). Their mean number of siblings was 1.47 ± 1.140, and the mean number of people living with them at home was 3.54 ± 1.353.

The general profile of the sample was, therefore, a nine-year-old child attending class every day, born in Spain, with Spanish parents and an older sibling, living with three other people at home.

### 3.2. Analysis of Participants’ Personal Autonomy

In terms of autonomy for performing hygiene, 80.8% of participants needed help with the hygiene aspects studied, primarily fingernail and toenail cutting (63.2% and 60.8% respectively), ear cleaning (41.6%), and hair washing (29.6%), while 8.4% need help for body washing. However, only 4.8% need help to clean their private parts, 4.8% need help to clean their teeth, and none require help with handwashing. For all these activities, children mainly receive help from their father or mother (Appendix A Table A1).

### 3.3. Analysis of Participants’ Knowledge of Hygiene

With regard to knowledge of general hygiene, a small percentage of children said they had been teased (had problems or were rejected) because they smelled or were dirty (4.8% and 0.8%, respectively), while 29.6% said it is important to be clean, so they are healthy, do not smell, are not rejected by their friends or punished at home, and to feel good. Most children knew that shower gel is used for body washing (94.4%) and shampoo for hair washing (92.8%). Interestingly, almost half the children (49.6%) did not know that you can still obtain head lice even if you wash your hair every day. With regard to hand hygiene, only 58.4% knew that washing your hands prevents diarrhoea. Most children of this age were aware that cleaning their teeth prevents cavities (96.0%). However, with regard to ear hygiene, only 46.4% knew that it is not good to clean their ears with cotton buds, and 21.6% said they did not know whether or not it is good. For foot hygiene, a little more than half the children knew that nails should be cut straight (57.6%), and 21.6% did not know how to cut their toenails. We, therefore, identified a lack of general health knowledge, especially with regard to ear and foot hygiene, although the children were well aware of body and oral hygiene (Table A1).

### 3.4. Analysis of Participants’ Performance of Hygiene Habits

The most common body and hair hygiene habits among the participants are: having a shower (82.4%), having a shower at night (61.6%), using shower gel, shampoo, and a sponge (61.6%), and possessing a towel and sponge for individual use (76.0% and 81.6%, respectively). In 65.6% of cases, participants wash their hair when they shower, most commonly, at night before they go to bed (61.6%). The most frequent hand hygiene habits are: washing hands more than three times a day (56.8%), always washing with soap (80.8%), always washing before meals (68.8%), washing hands after defecating (80.8%) and urinating (72.8%), and using a towel to dry their hands (92.0%). It was noteworthy that around 30% of participants do not correctly perform the habit of always washing their hands before eating or after urinating. Approximately 20% do not use soap to wash their hands, and the same percentage do not always wash their hands after defecating. The most frequent oral hygiene habits are: cleaning teeth with a toothbrush and toothpaste (41.6%) and using a manual toothbrush (64.0%) for individual use (99.2%). The average frequency of teeth cleaning is more than three times a day (40.8%) for one to three minutes (63.2%). Participants clean their teeth when they get up in the morning and at bedtime, and always after their main meals (12.8%), although the same percentage of participants do not clean their teeth after every meal. Although 56.8% of participants go to the dentist more than twice a year, it is noteworthy that 4.0% have never been to a dentist, and 14.4% have not been to one in the last year. In relation to when they should replace their toothbrush, 56.8% answered after three months or before if it wears out, although 23.2% said that they replace it only if it is worn out, and 12.0% said they do not know when they should replace it. With regard to ear hygiene habits, most participants use cotton buds for cleaning (81.6%) and 45.6% clean their ears two or three times a week. For intimate hygiene habits, most participants have been instructed by their parents and family members (55.2%), although 9.6% said that apart from family members, their doctor and a nurse also taught them. These habits are: washing their private parts after going to the toilet (42.4%) or while showering (24.8%), washing their private parts without washing the rest of their body (55.2%), or only washing when they shower or go to the toilet (44.8%). While 91.2% change their underwear every day, 8.8% do not have clean underwear every day. For foot hygiene, participants wash their feet without washing the rest of their body (81.6%) and do not always use the same footwear (72.0%) (Table A1).

### 3.5. Association between Sociodemographic Characteristics, Personal Autonomy, Hygiene Knowledge and/or Habits, and Type of Schooling

#### 3.5.1. Association between Participants’ Sociodemographic Characteristics and Type of Schooling

Table 1 show the associations between sociodemographic characteristics and type of schooling, indicating various differentiating characteristics between the two groups: children in PS are older than children in MS (9.88 and 8.96 years, respectively) (*p*-value 0.000). The country of birth of the child, father, and/or mother also shows differences, as children in PS were foreign and/or have a foreign father and/or mother, while children in MS were born in Spain, and most of their parents are Spanish (*p*-value 0.022, 0.000, 0.000, respectively). Children in PS typically have more siblings, and their siblings are older than them (*p*-value 0.000 and 0.001, respectively). Moreover, these children are more likely to have lived in children’s homes (*p*-value 0.038) or live with more people at home (*p*-value 0.000). We found no difference by gender in any of the sociodemographic characteristics studied. The text continues here.

#### 3.5.2. Association between Participants’ Personal Autonomy in Performing Hygiene and Type of Schooling

**Figure 2:**
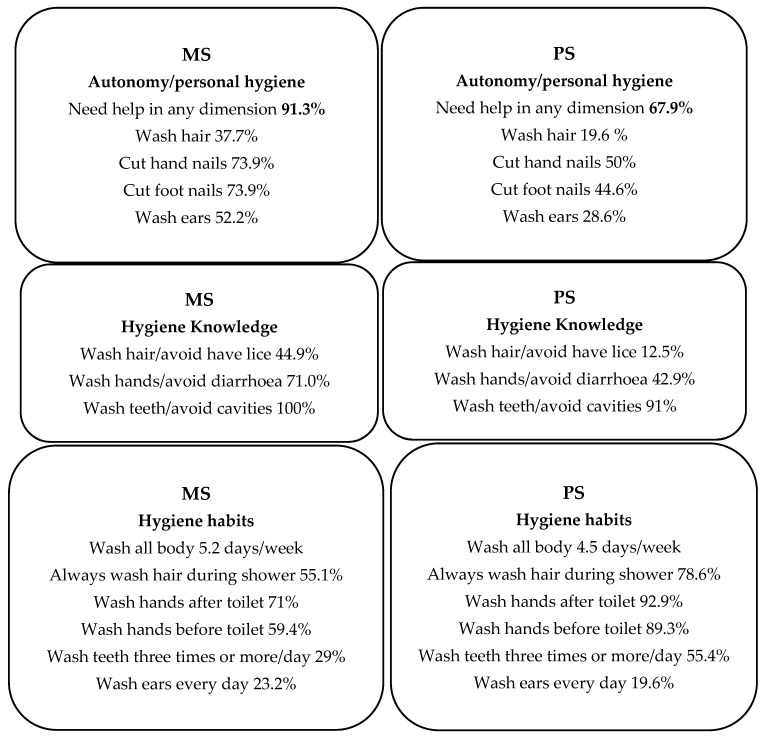
Graphical illustration of the results in the differences in the three dimensions analysed (personal autonomy, habits, and knowledge hygiene) related to the kind of schooling. MS: Mainstream Schooling; PS: Preferential Schooling; %: percentage.

Table 2 show the associations obtained for autonomy in the aspects of hygiene studied. Analysis of personal autonomy by type of schooling shows that children in PS say they receive less help in general (*p*-value 0.001) with hair washing (*p*-value 0.032), fingernail cutting (*p*-value 0.009), toenail cutting (*p*-value 0.001), and ear cleaning (*p*-value 0.010). The principal differences are included in Figure 2.

#### 3.5.3. Association between Participants’ Knowledge of Performing Hygiene and Type of Schooling

Table 3 show the associations identified regarding knowledge of the aspects of hygiene studied. Analysis of the association between knowledge and type of schooling shows differences in knowledge about whether hand washing prevents diarrhoea and whether cleaning your teeth helps to prevent cavities forming, indicating that children in PS have less knowledge about these aspects (*p*-value 0.006 and 0.016, respectively). The principal differences are included in Figure 2.

#### 3.5.4. Association between Participants’ Hygiene Habits and Type of Schooling

Analysis of the association between hygiene habits and type of schooling shows differences in the number of days per week participants wash their bodies, which is lower among children in PS, and the time of day for performing this habit (*p*-value 0.035 and 0.017, respectively). Differences were also observed in hair washing with every shower, performed more in this manner by children in PS (*p*-value 0.008), and the time of day for hair washing, performed in the evening by children in MS and at various times of day by children in PS (*p*-value 0.038). Hand washing showed differences in performance after defecating or urinating, as most children in PS indicated that they always wash their hands at these times (*p*-value 0.006 and 0.000, respectively). For oral habits, children in PS clean their teeth more times a day and use a manual toothbrush more frequently (*p*-value 0.008 and 0.002). Ear hygiene habits showed differences in the frequency of ear cleaning, as children in PS clean their ears two to three times a week while children in MS clean their ears every day. Intimate hygiene showed differences in the time of day for performing this activity, with children in PS washing their private parts after using the toilet and children in MS doing so when they shower (*p*-value 0.038). Differences were also observed in the person who taught the children to wash and clean themselves: in both cases, they were taught mainly by their parents or family members, although it is noteworthy that 16.2% of children in PS said they had taught themselves (Table 4). The principal differences are included in Figure 2.

A summary of the most relevant results regarding personal autonomy, habits, and hygiene knowledge in relation to the type of schooling is included in the graphical illustration in Figure 2.

### 3.6. Association between Participants’ Sociodemographic Characteristics, Personal Autonomy, Hygiene Knowledge and Habits, and Gender

#### 3.6.1. Association between Participants’ Sociodemographic Characteristics and Gender

Statistical analysis showed no differences between sociodemographic characteristics and gender.

#### 3.6.2. Association between Participants’ Autonomy in Personal Hygiene and Gender

Analysis of the data revealed statistically significant differences only in the need for help with hair washing (*p*-value 0.047), for which 64.9% of the girls and only 21.3% of the boys said they required help (Table 5).

#### 3.6.3. Association between Participants’ Knowledge of Personal Hygiene and Gender

Statistical analysis showed no differences between knowledge of personal hygiene and gender.

#### 3.6.4. Association between Hygiene Habits and Gender

Differences associated with hygiene habits by gender were identified in the manner and the frequency of body and hair washing. Although the shower is the predominant method chosen by both genders (88.5% for boys, 76.6% for girls), a significant difference was observed in using the bath for this purpose (21.8% for boys, 6.6% for girls) (*p*-value 0.007). Significant differences were also observed in the weekly frequency of hair washing (*p*-value 0.002), which was higher among boys than among girls (4.79 and 3.78 days, respectively). To answer the question regarding whether they always wash their hair when they shower, 85.2% of boys said yes, compared to only 46.9% of girls, with significant differences (*p*-value 0.000). In answer to the question of whether their sponge was for individual use, most girls said yes (95.3%), compared to 77.0% for boys, also with statistically significant differences (*p*-value 0.004) (Table 5).

## 4. Discussion

### 4.1. Sociodemographic Characteristics of Participants and Families

The sociodemographic profile of participants confirms the characteristics that necessitate differential schooling of the groups studied [9]. The age differences observed, showing that the group in PS is older, may be due to pupils having to repeat a year because of difficulties with the syllabus or adapting to life in Spain, as this group includes more foreign and Romani children. Moreover, some children in this group have had to live in children’s homes, further strengthening this line of thought (see Figure 2). We believe that educational care programs focused on multicultural education, student reception, attention to linguistic diversity and culture, as well as teacher training, can help these children and avoid the loss of the course (formation) and/or their integration into the educational system.

### 4.2. Participants’ Autonomy and Personal Hiygiene

Among the differences between the groups in need of help with personal hygiene, it is noteworthy that children in PS require less help with the hygiene habits addressed in the study (see Figure 2). This could be because they are more autonomous or do not ask their parents for help in their culture or ethnic group [16].

### 4.3. Participants’ Knowledge of Hygiene

The opposite occurs with the differences in the groups for knowledge of hygiene, as children in PS have less knowledge of hygiene habits (head lice/diarrhoea/tooth cavities) (See Figure 2). This indicates that although children in PS do not require help with their personal hygiene, they may need extra assistance to acquire the necessary knowledge, given that most knowledge of hygiene is learned in the first years of primary [7]. Moreover, these children are sometimes placed in a year that is too advanced for them or face a linguistic barrier in the classroom, hindering their acquisition of hygiene knowledge [17]. However, the difference between groups may also be due to knowledge that is not included in the syllabus, e.g., head lice, and we, therefore, support the findings of other authors who noted this lack in the Spanish school syllabus [1]. Children’s knowledge gaps could also be due to cultural reasons [18]. For all the results obtained, we believe that it is necessary to reinforce the knowledge about hygiene from school and, if necessary for cultural reasons, to facilitate the introduction of informative workshops for the relatives who are responsible for teaching children from an early age.

### 4.4. Participants’ Performance of Hygiene Habits

With regard to the differences in hygiene habits between the two groups studied, children in PS wash their body and ears fewer times a week, although they have a higher frequency of washing their hair when they shower, washing their hands after defecating or urinating, and washing their private parts after using the toilet. This could be because teachers at preferential schools focus on reinforcing these habits, necessitated by the personal situation of many of their pupils, whereas in mainstream schools, it is not considered necessary because children receive instruction at home. We believe that hygienic habits in children can still be improved, so they must be reinforced from school and the family.

The literature shows that the various components of hygiene have not been studied to the same extent or been considered by health authorities. The WHO and most researchers have addressed hand hygiene to prevent the spread of disease [19,20,21,22,23,24]. Oral hygiene has also been widely studied [25,26,27,28]. However, fewer studies have individually addressed other aspects of hygiene, such as hair and body washing, ear cleaning, and intimate hygiene [29,30,31,32,33]. Our results and those of other authors, however, reveal a lack of hygiene knowledge and habits, not only in hand washing but also in other aspects such as general hygiene and hygiene specific to foot care [34,35]. Our study provides new data concerning autonomy among children aged 8 to 11 years with regard to all aspects of personal hygiene. It is noteworthy that nearly all the participants are autonomous in teeth cleaning, possibly because the public sector has introduced numerous programs for child hygiene education, mainly in oral health [23], whereas they are more dependent for fingernail and toenail cutting. Because of this, even though we consider that the official compulsory education syllabus is adequate for the acquisition of hygiene skills [1], we agree with other authors [36] that more age-appropriate theory and practical sessions should be introduced, as constant repetition helps children to assimilate concepts and use age-appropriate techniques [3]. These measures will help children to gain knowledge and acquire general hygiene habits and should be implemented with interprofessional collaboration (teachers and health professionals).

## 5. Conclusions

Children from mainstream schooling (MS) wash their body and ears more and have less hygiene knowledge than children from preferential schooling (PS). However, children from PS wash their hair more when showering and their hands after using the toilet. These children from PS were also found to have more personal autonomy in general.

A high percentage of surveyed children aged 8 to 11 years (80.8%) require help to perform their personal hygiene, mainly for washing their bodies and cutting their fingernails and toenails.

Moreover, not all children perform personal hygiene activities correctly. Because of this, we consider it necessary to implement theory and practical workshops for the children who are starting to acquire personal hygiene habits autonomously in order to identify incorrect habits and increase their knowledge of hygiene, with particular reference to toenail cutting.

## Figures and Tables

**Table 1:** Association between sociodemographic variables and type of schooling.

Sociodemographic Variables		MS	PS	*p*-Value
Age		8.96	9.88	0.000
Country of birth of child	Spain	98.6%	87.5%	0.022
	Outside of Spain	1.4%	12.5%	
Country of birth of mother	Spain	100%	39.3%	0.000
	Outside of Spain	0.0%	60.7%	
Country of birth father	Spain	100%	48.2%	0.000
	Outside of Spain	0.0%	51.8%	
How many siblings do you have?		1.12	1.91	0.000
How many of your siblings are older than you?		0.54	1.0	0.001
How many people live with you?		3.17	3.98	0.001
How many siblings do you have?	None	20.3%	9.0%	0.000
	One sibling	58.0%	32.1%	
	Two siblings	17.4%	33.9%	
	Three or more siblings	4.3%	25.0%	
Have you ever lived in a children’s home?	Yes	0.0%	7.1%	0.038
	No	100%	92.9%	
How many people live with you?	Two people	18.8%	14.3%	0.000
	Three people	58.0%	26.8%	
	Four people	17.5%	26.8%	
	Five people	1.4%	16.1%	
	Six people	1.4%	7.1%	
	Seven or more people	2.9%	8.9%	

MS = Mainstream schooling, PS = Preferential schooling, % = percentage.

**Table 2:** Association between personal autonomy and type of schooling.

Variables Associated with Autonomy and Type of Schooling		MS	PS	*p*-Value
Help with some aspect	No	8.7%	32.1%	0.001
	Yes	91.3%	67.9%	
Help with hair washing	No	62.3%	80.4%	0.032
	Yes	37.7%	19.6%	
Help with fingernail cutting	No	26.1%	50%	0.009
	Yes	73.9%	50%	
Help with cutting toenails	No	26.1%	55.4%	0.001
	Yes	73.9%	44.6%	
Help with ear cleaning	No	47.8%	71.4%	0.010
	Yes	52.2%	28.6%	

MS = Mainstream schooling, PS = Preferential schooling, % = percentage.

**Table 3:** Association between knowledge of hygiene and type of schooling.

Variables Associated with Knowledge About Hygiene and Type of Schooling		MS	PS	*p*-Value
If you wash your hair every day, can you still obtain head lice?	Yes	44.9%	12.5%	0.000
	No	40.6%	60.7%	
	Do not know	14.5%	26.8%	
Does washing your hands stop you from developing diarrhoea?	Yes	71.0%	42.9%	0.006
	No	13.1%	23.2%	
	Do not know	15.9%	33.9%	
Does cleaning your teeth help to stop you from developing holes in them?	Yes	100%	91.0%	0.016
	No	0.0%	5.4%	
	Do not know	0.0%	3.6%	

MS = Mainstream schooling, PS = Preferential schooling, % = percentages.

**Table 4:** Association between hygiene habits and type of schooling.

Variables Associated with Hygiene Habits and Type of Schooling		MS	PS	*p*-Value
How many times have you washed your body in the last 7 days?		5.20 days	4.5 days	0.035
What time of day do you wash your body?	At night	69.6%	51.8%	0.017
	Some days in the morning and some days in the afternoon or at night	26.2%	30.4%	
	In the afternoon	1.4%	14.2%	
	When I get up in the morning	1.4%	3.6%	
	When I get up and when I go to bed	1.4%	0.0%	
Do you always wash your hair when you have a shower?	Yes	55.1%	78.6%	0.008
	No	44.9%	21.4%	
What time of day do you wash your hair?	At night	72.5%	48.2%	0.038
	Some days in the morning and other days in the afternoon or at night	21.7%	35.7%	
	In the afternoon	2.9%	7.2%	
	When I get up in the morning	2.9%	8.9%	
	When I get up and when I go to bed	0.0%	0.0%	
Do you wash your hands after doing a pooh?	Always	71.0%	92.9%	0.006
	Nearly always	14.6%	7.1%	
	Hardly ever	7.2%	0.0%	
	Never	7.2%	0.0%	
Do you wash your hands after doing a pee?	Always	59.4%	89.3%	0.000
	Nearly always	23.2%	10.7%	
	Hardly ever	5.8%	0.0%	
	Never	11.6%	0.0%	
	Nearly always	1.4%	8.9%	
	I do not dry my hands	1.4%	1.8%	
	Air dryer	0.0%	1.8%	
	Towel and paper	0.0%	1.8%	
How many times did you clean your teeth yesterday?	None	7.2%	0.0%	0.008
	One	21.8%	14.2%	
	Two	42.0%	30.4%	
	Three or more times	29.0%	55.4%	
What type of toothbrush do you use?	Manual	52.2%	78.6%	0.002
	Electric	37.7%	21.4%	
	Both	10.1%	0.0%	
How many times have you cleaned your ears in the last 7 days?	Every day	23.2%	19.6%	0.034
	Two or three times	36.2%	57.2%	
	One	23.2%	19.6%	
	None	17.4%	3.6%	
When do you wash your private parts?	After using WC	29.0%	58.8%	0.038
	When I have a shower	30.7%	17.9%	
	AB	11.6%	10.7%	
	GU	4.3%	5.4%	
	After using WC, GU, AB	4.3%	0.0%	
	After using WC and in the shower	4.3%	3.6%	
	GU, AB	2.9%	1.8%	
	Never	1.4%	1.8%	
	Other times: when I have not sweated, I wash my private parts only	8.7%	0.0%	
	When there are no wet wipes	1.4%	0.0%	
	After using WC and AB	1.4%	0.0%	
Who taught you how to wash and clean yourself?	PF	59.7%	50.0%	0.000
	PF, DN	8.7%	10.6%	
	PF, T	14.4%	1.8%	
	S	0.0%	16.1%	
	PF, T, DN	4.3%	8.9%	
	PF, T, DN, S	4.3%	3.6%	
	PF, T, S	5.8%	0.0%	
	T	0.0%	1.8%	
	PF, RTI, DN, CHS	0.0%	1.8%	
	PF, DN, S	1.4%	0.0%	
	PF, S	0.0%	5.4%	
	PF, RTI, T	1.4%	0.0%	

MS = Mainstream schooling, PS = Preferential schooling, % = percentage, WC = Toilet, AB = At bedtime, GU = When I get up, PF = Parents and family members, DN = Doctor and nurse, T = Teacher, S = I taught myself, RTI = radio, tv, internet; CHS = Children’s home staff.

**Table 5:** Association between sociodemographic variables, autonomy, knowledge and habits, and gender.

Variables Associated with Autonomy in Hygiene and Gender		Male	Female	*p*-Value
Does someone help you wash your hair?	Yes	21.3%	64.9%	0.047
	No	78.7%	35.1%	
Variables associated with hygiene habits and gender		Male	Female	*p*-value
How do you usually wash yourself?	Shower	88.5%	76.6%	0.007
	Bath	6.6%	21.8%	
	Sponge and towel	0.0%	1.6%	
	Shower and bath	4.9%	0.0%	
Do you always wash your hair when you have a shower?	Yes	85.2%	46.9%	0.000
	No	14.8%	53.1%	
How many times have you washed your hair in the last seven days?		4.79	3.78	0.002
Is your sponge for your use only?	Yes	77.0%	95.3%	0.004
	No	23.0%	4.7%	

% = percentage.

## Data Availability

The data used to support the findings of this study are available from the firth author or the corresponding author upon request.

## Appendix A

**Table A1:** Sociodemographic characteristics of participants and their families. Analysis of hygiene autonomy, knowledge, and habits of participants in the aspects of hygiene studied.

Characteristics of Participants. Sociodemographic Data		
Study variable	Mean (standard deviation)	Mean (standard deviation)
Mean age of all participating children (years)	Total 9.37 (±1.044)	On arriving in Spain 6.0 (±3.786)
Mean age by gender (years)	Male 9.33 (±0.691)	Female 9.41 (±1.321)
Age of children by type of schooling	MS 8.96 (±1.077)	PS 9.88 (±0.740)
Study variable	*n* (%)	*n* (%)
Type of schooling	MS 69 (55.2%)	PS 56 (44.8%)
Gender	Female 64 (51.2%)	Male 61 (48.8%)
Country of birth of children	Spain 117 (93.6%), Other countries 8 (6.4%)	
Country of birth mother	Spain 91 (72.8%), Morocco 21 (16.8%), Other countries (Peru, Romania, France, Germany, Nicaragua) 13 (10.4%)	Spain 96 (72.8%) Outside of Spain 29 (27.2%)
Country of birth father	Spain 96 (76.8%), Morocco 20 (16.0%), Other countries (Nicaragua, Peru, Egypt) 9 (7.2%)	Spain 96 (76.8%) Outside of Spain 34 (23.2%)
How many siblings do you have?	No sibs 19 (15.2%), 1 sib 58 (46.4%), 2 sibs 31 (24.8%), 3 or more sibs 17 (13.6%)	Mean number of sibs 1.47 (±1.140)
Have you ever lived in a children’s home?	No 121 (96.8%), Yes 4 (3.2%)	
How many older siblings do you have?	No older sibs 68 (54.4%), 1 sib 36 (28.8%), 2 sibs 10 (8.8%), 3 or more sibs 10 (8.0%)	Mean number of older sibs 0.74 (±1.054)
How many people live with you?	2 p 21 (16.8%), 3 p 55 (44.0%), 4 p 27 (21.6%), 5 p 10 (8.0%), 6 p 5 (4.0%), More than 7 p 7 (5.6%)	Mean number of people living with the child 3.54 (±1.353)
Days attending class	Every day 124 (99.2%), 3–4 days a week 1 (0.8%)	
Analysis of Participants’ Autonomy in Personal Hygiene		
Study variable	*n* (%)	*n* (%)
Do you need help with hygiene in any of the aspects of the study?	Yes 101 (80.8%), No 24 (16.8%)	
Does anyone help you wash your body?	No 102 (81.6%), MF 20 (16%), My father, mother or older sib 2 (1.6%), My sib 1 (0.8%)	No 102 (81.6%), Yes 23 (8.4%)
Does anyone help you wash your hair?	No 88 (70.4%), MF 23 (26.4%) My sib 2 (1.6%), My father, mother or older sib 1 (0.8%), My father, mother or by myself 1 (0.8%)	No 88 (70.4%), Yes 37 (29.6%)
Does anyone help you wash your hands?	No 125 (100%)	
Does anyone help you cut your fingernails?	MF 78 (62.4%), No 29 (23.2%) No—I bite them 15 (12.0%), No—I never cut them 2 (1.6%), Others (my grandfather) 1 (0.8%)	Yes 79 (63.2%), No 46 (36.8%)
Does anyone help you clean your teeth?	No 124 (99.2%), MF 1 (0.8%)	No 124 (99.2%), Yes 1 (0.8%)
Does anyone help you cut your toenails?	MF 75 (60%), No 47 (37.6%) No—I never cut them 2 (1.6%), Others (my grandfather) 1 (0.8%)	Yes 76 (60.8%), No 49 (39.2%)
Does anyone help you clean your ears?	s 69 (55.2%), MF 52 (41.6%), No—I never clean them 4 (3.2%)	No 73 (58.4%), Yes 52 (41.6%)
Does anyone help you wash your private parts?	No 119 (95.2%), Yes 6 (4.8%)	
Does anyone help you wipe your bottom after you do a pooh?	s 118 (94.4%), MF 6 (4.8%), No—I never wipe my bottom 1 (0.8%)	No 119 (95.2%), Yes 6 (4.8%)
Knowledge of General Hygiene		
Do other children ever tease you because you smell?	Never 119 (95.2%), Occasionally 5 (4.0%) Often 1 (0.8%)	No 119 (95.2%), Yes 6 (4.8%)
Do other children ever tease you because you’re dirty?	No 124 (99.2%), Occasionally 1 (0.8%)	No 124 (99.2%), Yes 1 (0.8%)
Why is it important to be clean?	TBH, NS, NRF, NPH, FG 37 (29.6%), TBH, NS, NRF, FG 29 (23.2%), TBH, NS, FG 20 (16.0%), TBH 7 (5.6%), TBH, NS 6 (4.8%), TBH, FG 5 (4.0%), NS, FG 4 (3.2%), TBH, NS, NPH, FG 3 (2.4%), NS 3 (2.4%), NS, FG 3 (2.4%), Other options <2% 8 (6.4%)	
If you wash your hair every day, can you still obtain head lice?	No 62 (49.6%), Yes 38 (30.4%), Do not know 25 (20.0%)	
What is shampoo used for?	WH 116 (92.8%), WS 7 (5.6%), WS, WH, Wh 1 (0.8%), Wh 1 (0.8%)	Correct use 116 (92.8%), Incorrect use 9 (7.2%)
What is shower gel for?	WS 118 (94.4%), WH 6 (4.8%), WS, WH, Wh 1 (0.8%)	Correct use 118 (94.4%), Incorrect use 7 (5.6%)
Does washing your hands stop you from developing diarrhoea?	Yes 73 (58.4%), No 22 (17.6%), Do not know 30 (24.0%)	
Does cleaning your teeth help to stop you from developing holes in them?	Yes 120 (96.0%), No 3 (2.4%), Do not know 2 (1.6%)	
Do you know that toenails should be cut straight?	Yes 72 (57.6%), No 29 (23.2%), Do not know 24 (19.2%)	
Do you think it’s right to clean your ears with cotton buds?	Yes 58 (46.4%), No 40 (32.0%), Do not know 27 (21.6%)	
Body and Hair Hygiene Habits		
How do you usually wash yourself?	Shower 103 (82.4%), Bath 18 (14.4%), Other options (sponge and towel/shower or bath) 4 (3.2%)	
How many times have you washed your body in the last 7 days?	Seven times 40 (32.0%), Four times 27 (21.6%), Three times 18 (14.4%), Five times 19 (15.2%), Six times 10 (8.0%), Two times 5 (4.0%), One time 4 (3.2%), No times 2 (1.6%)	
What time of day do you wash your body?	BB 77 (61.6%), SDMODAE 35 (28.0%), AHS 9 (7.2%), M 3 (2.4%), GU and AB 1 (0.8%)	
Do you always wash your hair when you have a shower?	Yes 82 (65.6%), No 43 (34.4%)	
How many times have you washed your hair in the last 7 days?	Seven times 28 (22.4%), Three times 25 (20.0%), Four times 26 (20.8%), Five times 15 (12.0%), Two times 15 (12.0%), Six times 8 (6.4%), One time 6 (4.8%), No times 2 (1.6%)	
What time of day do you wash your hair?	BB 77 (61.6%), SDMODAE 35 (28.0%), AHS 6 (4.8%), GUM 7 (5.6%)	
What do you use for showering or having a bath?	SG, S, Sp 82 (65.6%), SG, S, So, Sp 17 (13.6%), SG, S 6 (4.8%), SG, S, Sp, BoB 6 (4.8%), SG, S, So, Sp, BoB 3 (2.4%), Other options 11 (8.8%)	
Is your sponge for your use only?	Yes 102 (81.6%), No 14 (11.2%), I do not use one 6 (4.8%), Do not know 3 (2.4%)	Yes 108 (86.4%), No 17 (13.6%)
Is the towel you dry yourself with for your use only?	Yes 95 (76.0%), No 25 (20.0%), I do not use one 1 (0.8%), Do not know 4 (3.2%)	Yes 96 (76.8%), No 29 (23.2%)
Hand Hygiene Habits		
How many times did you wash your hands yesterday?	More than three 71 (56.8%), Three times 28 (22.4%), Two times 17 (13.6%), One time 8 (6.4%), No times 1 (0.8%)	
Do you use soap when you wash your hands?	Always 101 (80.8%), Nearly always 21 (16.8%), Hardly ever 2 (1.6%), Never 1 (0.8%)	
Do you wash your hands before every meal?	Always 85 (68.0%), Nearly always 33 (26.4%), Hardly ever 6 (4.8%), Never 1 (0.8%)	
Do you wash your hands after doing a pooh?	Always 101 (80.8%), Nearly always 14 (11.2%), Hardly ever 5 (4.0%), Never 5 (4.0%)	
Do you wash your hands after doing a pee?	Always 91 (72.8%), Nearly always 22 (17.6%), Hardly ever 4 (3.2%), Never 8 (6.4%)	
What do you dry your hands with after you wash them at home?	Towel 115 (92.0%), Paper 6 (4.8%), I do not dry my hands 2 (1.6%), Air dryer 1 (0.8%), Towel and paper 1 (0.8%)	
Oral Hygiene Habits		
How many times did you clean your teeth yesterday?	Three or more times 51 (40.8%), Two times 46 (36.8%), One time 23 (18.4%), None 5 (4.0%)	
What time of day do you clean your teeth?	GUM, ASMBNA, BB 16 (12.8%), GUM, AAMM, BB 16 (12.8%), GUM, AAMM, ASMBNA, BB 14 (11.2%), AAMM, BB 13 (10.4%), AAMM 11 (8.8%), BB 8 (6.4%), AAMM, ASMBNA, BB 8 (6.4%), ASMBNA, BB 8 (6.4%), GUM, BB 7 (5.6%), GUM, AAMM 4 (3.2%), OWR 4 (3.2%), ASMBNA 3 (2.4%), Other options 13 (10.4%)	
How much time do you spend cleaning your teeth?	1 to 3 min 79 (63.2%), Do not know 19 (15.2%), Not much, <1 min 18 (14.4%), A long time >3 min 8 (6.4%), I do not clean my teeth 1 (0.8%)	
What do you use to clean your teeth with?	Toothbrush and toothpaste 52 (41.6%), Toothbrush, toothpaste, mouthwash 36 (28.8%), Toothbrush, toothpaste, mouthwash, dental floss 14 (11.2%), Toothbrush, toothpaste, toothpick 6 (4.8%), Toothbrush, toothpaste, dental floss 5 (4.0%), Toothbrush, toothpaste, mouthwash, toothpick 4 (3.2%), Other options 8 (6.4%)	
Is your toothbrush for your use only?	Yes 124 (99.2%), No 1 (0.8%)	
What type of toothbrush do you use?	Manual 80 (64.0%), Electric 38 (30.4%), Both 7 (5.6%)	
When should you throw away your toothbrush?	After 3 months unless it wears out earlier 71 (56.8%), Only if it wears out 29 (23.2%), Do not know 15 (12.0%), After 4 months or longer 5 (4.0%), Every year at the start of the new school year 3 (2.4%), I never throw it out 2 (1.6%)	
How many times have you been to the dentist in the last year?	One or more 71 (56.8%), One 31 (24.8%), None this year 18 (14.4%), I’ve never been to the dentist 5 (4.0%)	
Foot Hygiene Habits		
Have you ever washed your feet without washing the rest of your body?	Yes 87 (69.6%), No 38 (30.4%)	
Do you always wear the same pair of shoes?	No 90 (72.0%), Yes 35 (28.0%)	
Ear Hygiene Habits		
How many times have you cleaned your ears in the last 7 days?	Two or three 57 (45.6%), Every day 27 (21.6%), One 27 (21.6%), Never 14 (11.2%)	
What do you clean your ears with?	A cotton bud 102 (81.6%), Water 7 (5.6%), I do not clean my ears 5 (4.0%), My finger 3 (2.4%), A seawater spray 3 (2.4%), Cotton bud and seawater 3 (2.4%), Pointed object (pencil, pen, key) 2 (1.6%), Pointed object and finger 1 (0.8%)	
Intimate Hygiene Habits		
Have you ever washed your private parts without washing the rest of your body?	Yes 69 (55.2%), No, I wash my private parts only when I have a shower or bath 56 (44.8%)	
When do you wash your private parts?	After using WC 53 (42.4%), When I have a shower 31 (24.8%), AB 14 (11.2%), GU 6 (4.8%), After WC and in the shower 5 (4.0%), After WC, GU, AB 3 (2.4%), GU, AB 3 (2.4%), Other options 10 (8%)	
Do you put on clean underwear every day?	Yes 114 (91.2%), Every 2–3 days 9 (7.2%), Every 3 days or more 2 (1.6%)	Yes 114 (91.2%) No 11 (8.8%)
Who taught you to wash and clean yourself?	PF 69 (55.2%), PF, DN 12 (9.6%), PF, T 10 (8.0%), s 9 (7.2%), PF, P, DN 8 (6.4%), PF, T, DN, s 5 (4.0%), PF, P, s, 3 (2.4%), Other options 9 (7.2%)	

MS = Mainstream schooling, PS = Preferential schooling, % = Percentage, Sib = Sibling, p = Person/people, MF = Mother or father, s = I taught myself, TBH = To be healthy, NS = Not smell, NRF = Not be rejected by friends, NPH = Not be punished at home, FG = Feel good, WH = Wash hair, WS = Wash skin, Wh = Wash hands, BB = Before I go to bed at night, SDMODAE = Some days in the morning and other days in the afternoon or at night, AHS = In the afternoon when I get home from school, M = In the morning, GU = When I get up, AB = At bedtime, GUM = When I get up in the morning, SG = Shower gel, S = shampoo, Sp = Sponge, So = Soap, BoB = Bucket or bowl, ASMBNA = After some meals but not all, AAMM = always after main meal, OWR = Only when I remember, WC = Toilet, PF = My parents and family members, DN = Doctor and nurse, T = Teacher.
